# Supplementary material for: Order–disorder and ionic conductivity in calcium nitride-hydride
Source: Nat Commun. 2023 Jul 20;14:4389. doi: 10.1038/s41467-023-40025-2 (PMC10359262; doi:10.1038/s41467-023-40025-2)
Supplement: Supplementary file 1 — Supplementary Information [file 41467_2023_40025_MOESM1_ESM.docx]

Supplementary Information

**Order-Disorder and Ionic Conductivity in Calcium Nitride-Hydride**

Irvine et al.


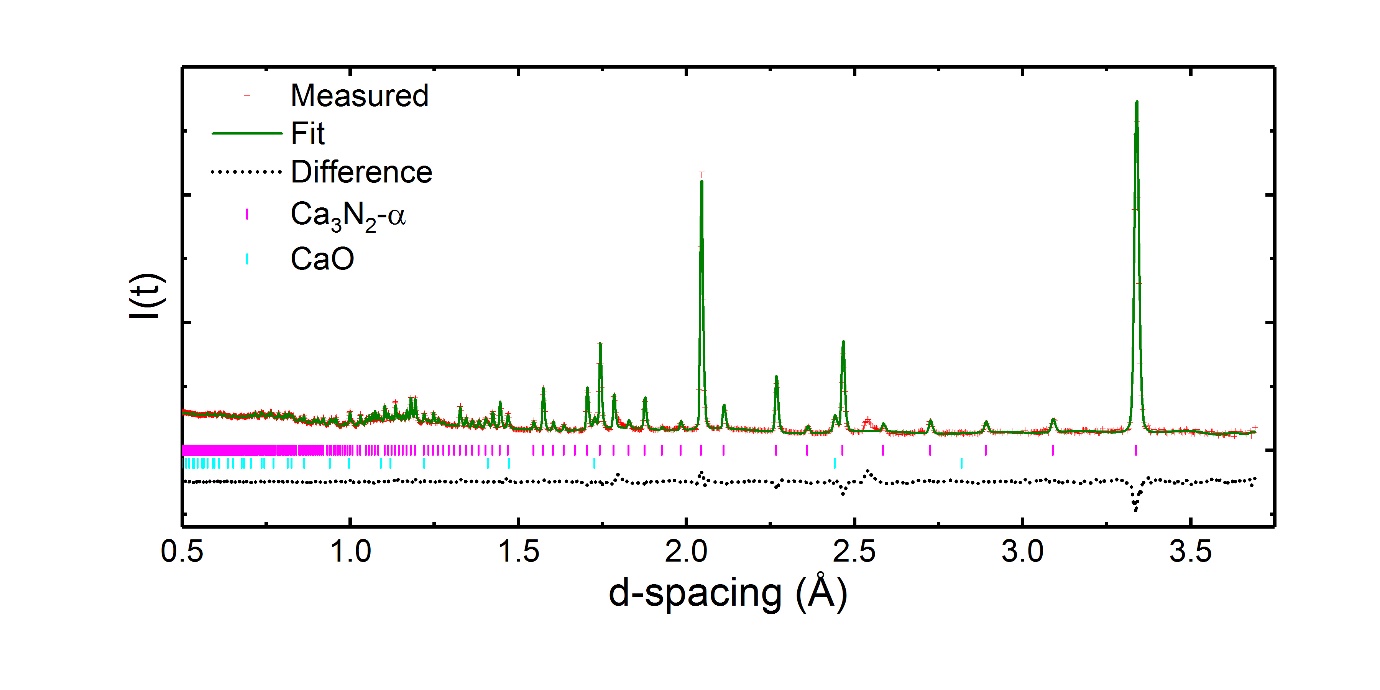


Supplementary Figure 1: α-Ca_3_N_2_ NPD pattern and refinement at 600 ^o^C. Data were collected on Polaris^1^ over approximately an hour. Two phases are indexed, the nitride, and CaO. The phase fraction of the oxide remained constant across the isotherm measurements, and the doping experiments (~10%). There is a further set of indexed peaks that also remained unchanged for the duration of the experiment, using the relative positions of the peaks, it was determined that these peaks corresponded to austenitic steel, likely from the sample holder itself. The Ca_3_N_2_ and CaO phase models were based off work published by Reckeweg et al and Shen et al.^2,3^. Structural Refinement conducted in GSAS.

Supplementary Table 1: Refinement results for α-Ca_3_N_2_. Results agree well with published results^2^. See Supplementary Figure 1 for further details.


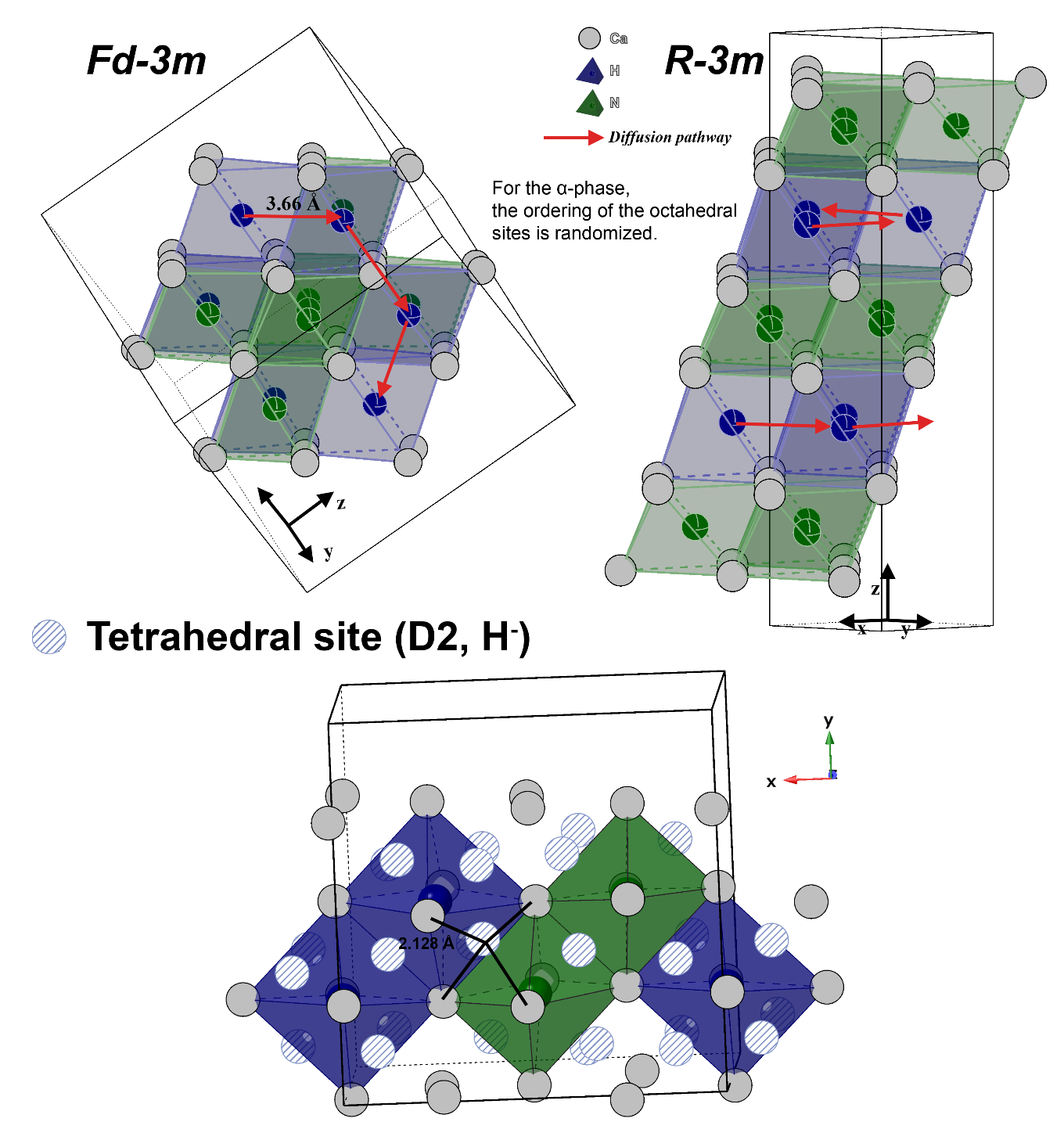


Supplementary Figure 2: Lattice ordering in the *Fd-3m* and *R-3m* space groups. Both space groups have a slightly distorted Ca^2+^ FCC arrangement. The difference is in the ordering of the octahedral sites surrounding the Ca^2+^ FCC: the *R-3m* space group results in layers of H^-^ and N^3-^ while the *Fd-3m* has a more complex arrangement based on the d glide plane symmetry. The *R-3m* space group results in a 2D diffusion pathway for H^-^ ions while the *Fd-3m* is 3D. Furthermore, a loss in ordering of the octahedral sites results in the *Fm-3m* structure (see Supplementary Figure 2). The position of the secondary tetrahedral site (D2, *48f*) is for the $\beta$-phase is also shown.

The *Fd-3m* and *R-3m* space groups are related to the rock-salt structure (*Fm-3m*). In both cases, the dramatically larger lattice structure associated with these space groups is a result of ordering of the octahedral positions due to the presence of two distinct anionic species. In the *Fd-3m* structure the octahedral positions form a 3D array based on the diamond glide plane symmetry operator (d), while the *R-3m* space group orders the octahedral species into layers. A randomized assignment of the octahedral species for both space groups would be equivalent to using the *Fm-3m* space group with mixed occupancy of the octahedral position. This is exactly what occurs with α-Ca_2_NH; the anionic species randomly occupy octahedral positions resulting in the phase being modelled with the *Fm-3m* space group rather than the larger high symmetry *Fd-3m* of $\beta$-Ca_2_NH. Supplementary Figure 2 shows the relationship between these space groups. The next figure (Supplementary Figure 3) gives a flow diagram how the site assignment and relative lattice sizes change between the super rock-salt structure and the rock-salt structure.


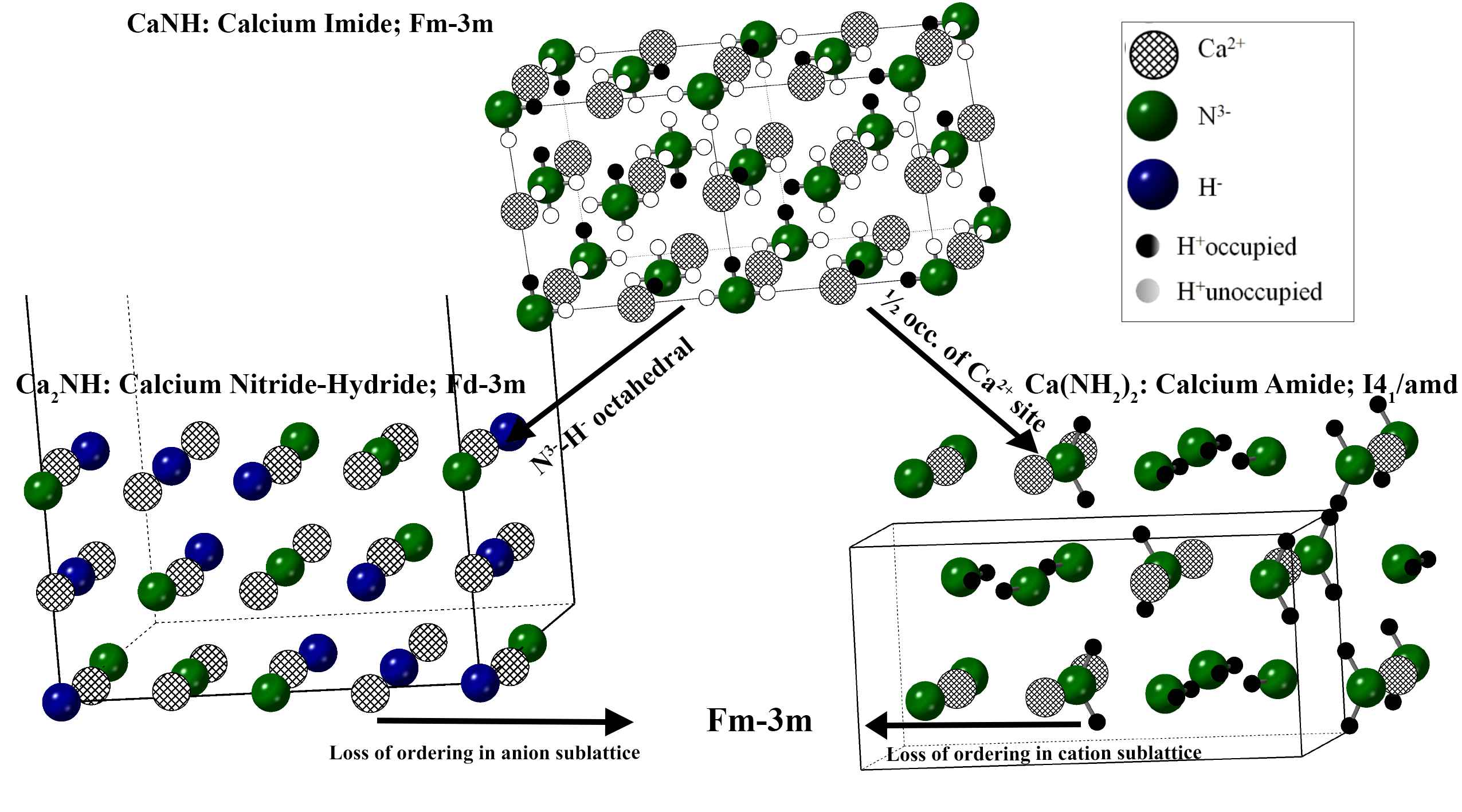


Supplementary Figure 3: The relationship between the structures of imide, amide, and nitride-hydride species of alkaline earth medals.


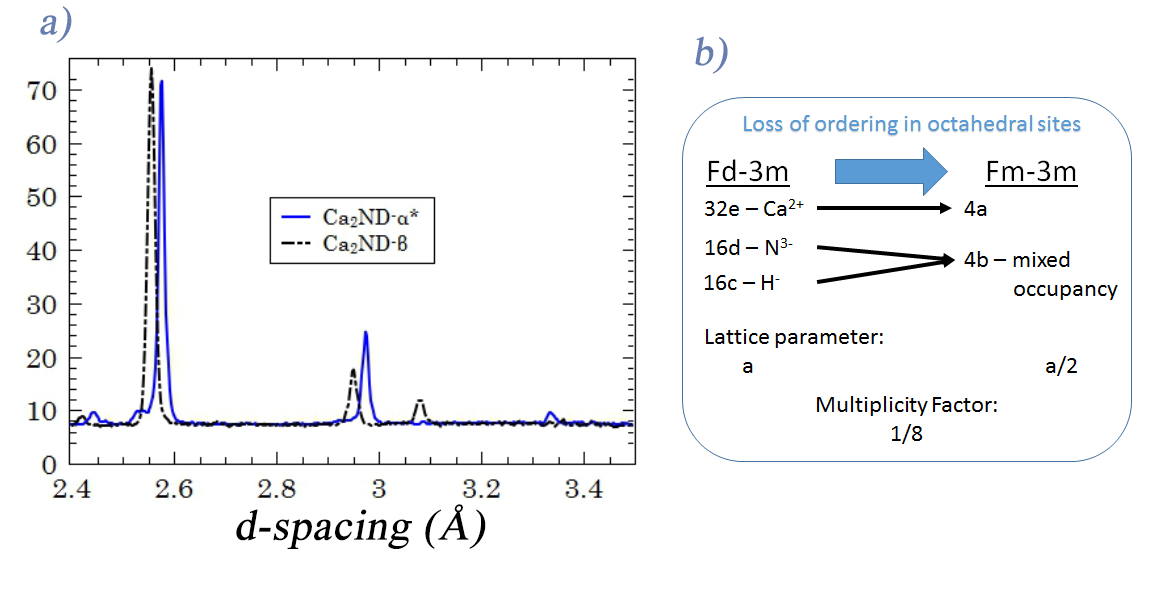


Supplementary Figure 4: A chart that shows the relationship between the imide (*Fm-3m*, rocksalt structure) and the nitride-hydride (*Fd-3m*) structures.

| Authors | Reaction | Structure | Lattice parameter: | Secondary anionic species |
| --- | --- | --- | --- | --- |
| Brice et al^4^ | CaH_2_+Ca_3_N_2_ →2Ca_2_NH | *Fd-3m* | 10.13 | none |
| Kitano et al^5^ | Ca_2_Ne^-^+½H_2_ →Ca_2_NH | *Fd-3m* | 10.13 | none |
| Verbraeken et al^6^ | 2Ca_(m)_+½N_2_+½H_2_ →Ca_2_NH | *R-3m* and *Fd-3m* | 10.16 | imide |
| Reckeweg and DiSalvo^7^ | Ca_3_N_2_+(1-x)Ca_(m)_+ xCaH_2_ → 2Ca_2_NH_x_ | *Fd-3m* | 10.135 | Ca2N |
| This work α- | 2Ca_3_N_2_+1½H_2_ → 3Ca_2_NH +½N_2_ | *Fm-3m* | 10.22-10.12 | imide |
| This work β- | 2CaH_2_+½N_2_ → Ca_2_NH+1½H_2_ | *Fd-3m* | 10.135 | none |

Supplementary Table 2: Summary of previous published synthesis route and structure for Ca_2_NH. The phase can be made from the electride (the same as the subnitride, Ca_2_N), the hydride, the nitride, pure calcium metal, or a mixture of these. Note, that Kitano et al did not refine their crystal structure. Verbraeken et al improved their quality of fit using the *R-3m* space group. Here, we report their *Fd-3m* space group refinement for comparison sake. Reckeweg and DiSalvo showed that with decreasing CaH_2_ precursor, that the lattice parameter of the resulting nitride-hydride phase increased. This result matches with the increase in lattice parameter we see for the α-phase reported in this paper.


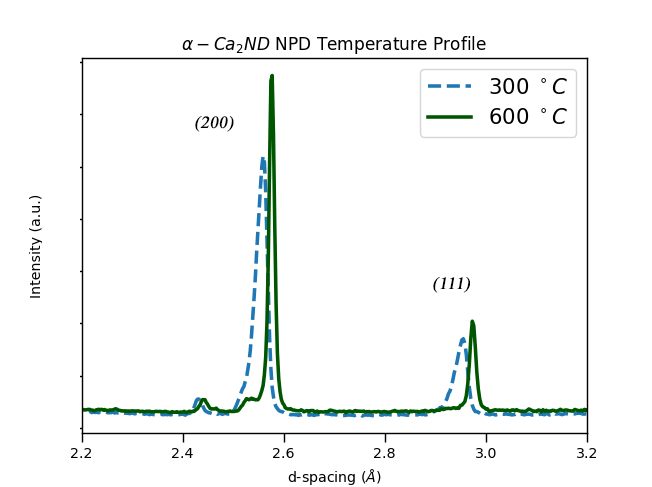


Supplementary Figure 5: Comparison between NPD pattern of $\alpha$-Ca_2_NH at 600 and 400 ^o^C collected on Polaris Diffractometer^1^. The development of broad asymmetric peaks upon cooling is characteristic of N-H systems with secondary anion species^6,8^.

Supplementary Table 3: Additional fitting statistics for the patterns from figure 1 and table 1.


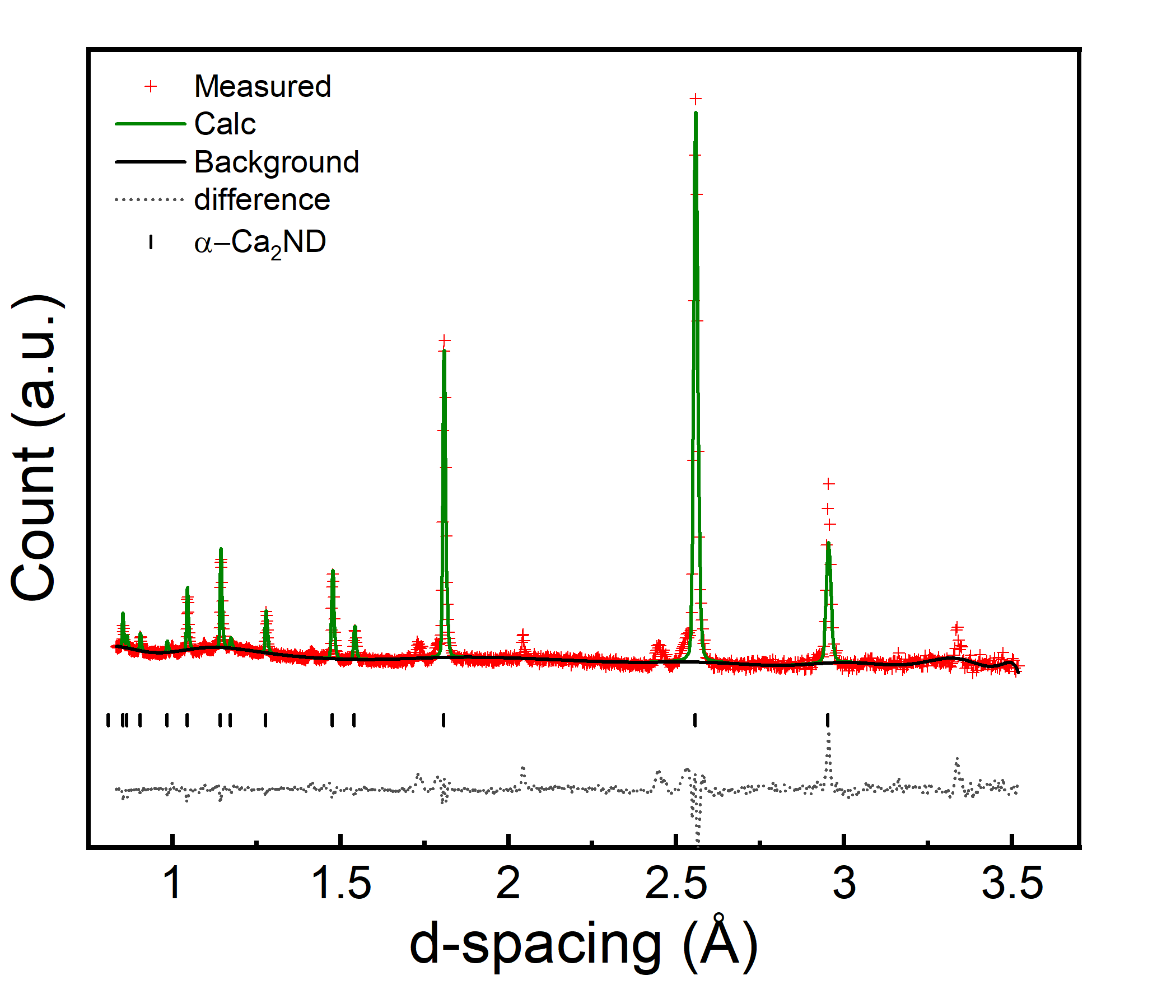


Supplementary Figure 6: Neutron diffraction pattern (Polaris, ISIS^1^) and refinement for $\alpha$-Ca_3_N_2_ reacted with D_2_ at 450 ^o^C. Structural refinement was done in GSAS. The result shows that the $\alpha$-Ca_2_ND phase is also formed at lower temperatures. The pattern was fit using the $\alpha$-Ca_2_ND reported in this paper with a lattice parameter of 5.11081(17) Å.

Supplementary Table 4: Refinement results for $\alpha$-Ca_3_N_2_ reacted with D_2_ at 450 ^o^C. Refinement conducted in GSAS. Data collected on Polaris Diffractometer at ISIS^1^. Model used was 600 ^o^C data refinement result given in main text.


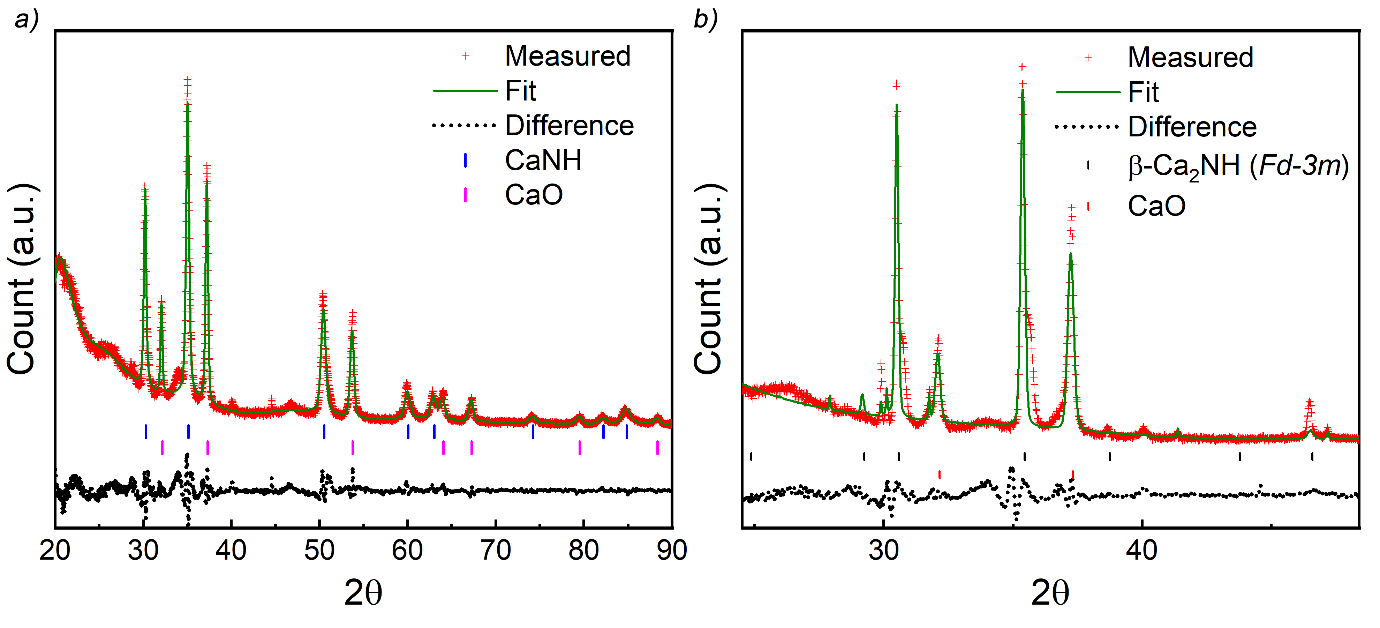


Supplementary Figure 7: XRD Patterns of $\alpha$-phase (a) and $\beta$-phase (b) after they were exposed to H_2_ gas at 800 ^o^C for 6 hrs. The $\alpha$-phase has been entirely converted to calcium imide (CaNH), while the $\beta$-phase shows now shows prominent shoulders associated with the presence of secondary anionic sites (such as imide or amide species). The pattern was collected using a PANalytical Empyrean with Cu K$\alpha$_1_ radiation between 20-90^o^ in .02^o^ increments. The data were collected over the course of an hour.

Supplementary Table 5: XRD Refinement data for α-phase and $\beta$-phase heat treated at 800 ^o^C in flowing H_2_ gas in Ar (5%). The structural refinements used Brice et al., Sichla et al., and Shen et al. for Ca_2_NH, CaNH and CaO respectively^3,4,9^. Note that the lattice parameter of the resulting calcium imide phase is significantly smaller than the published structure from Sichla et al. (5.143 Å). This difference suggests that the phase my still contain nitride-hydride species.


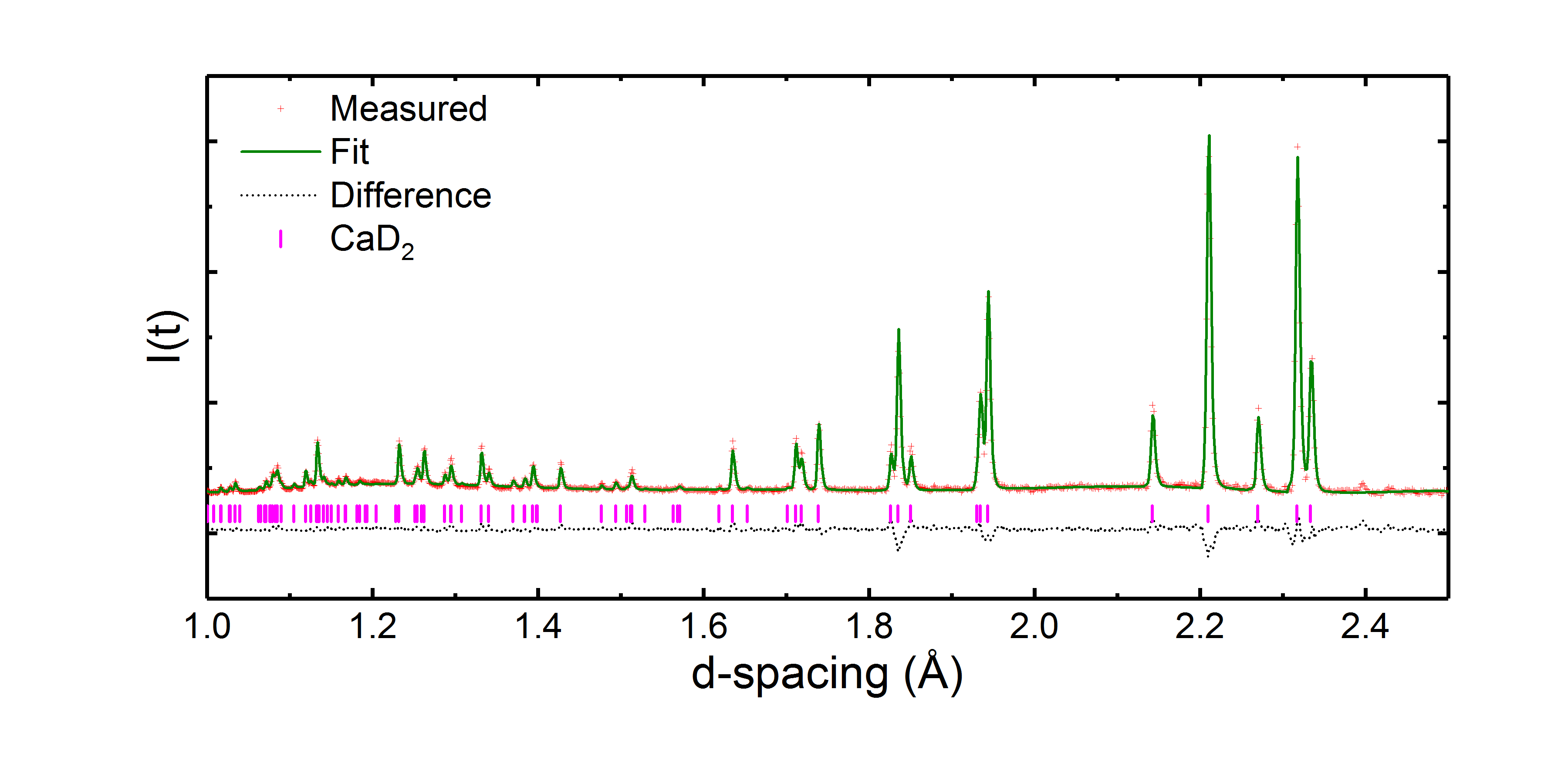


Supplementary Figure 8: CaD_2_ diffraction pattern collected on Polaris^1^ at 600 °C. The refinement shows a pure calcium hydride(deuteride phase). The tabulated fit parameters are shown in Supplementary Table 5. The structural refinement was based on the structure published by Alonso et al.^10^. The structural refinement was conducted using GSAS.


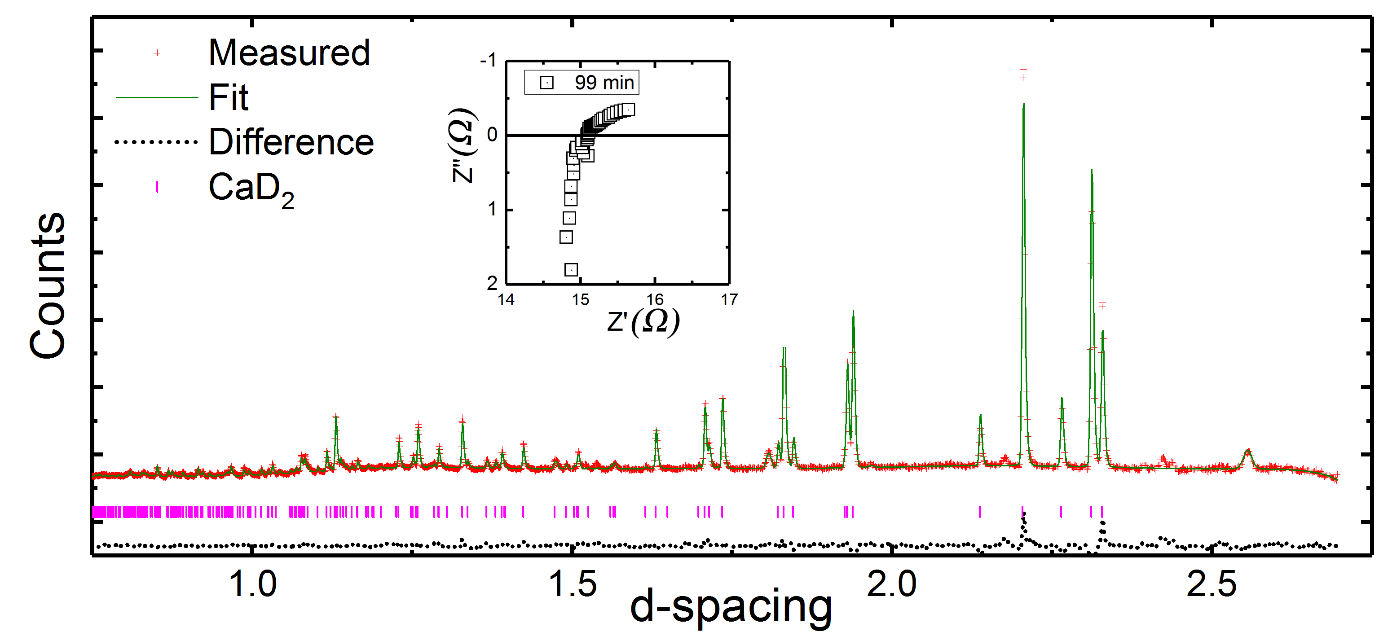


Supplementary Figure 9: Pattern and fit for the first 25 datasets summed from doping CaD_2_ with N_2_. Data were collected on Polaris Diffractometer^1^. Notice the small peaks at ~2.7 Å, ~2.4 Å, and ~2.2 Å. These are the nascent peaks of the $\beta$-Ca_2_ND phase that only begin to appear at the 25th scan. The EIS data show an impedance spectrum collected 99 minutes into the experiment. Both phases were fit using the *Pnma* space group. Calcium occupancy was fixed at 1. Supplementary Table 5 gives the Rietveld refinement fitting parameters and statistics. GSAS was used for the structural refinement.

Supplementary Table 6: NPD refinement results for pure CaD_2_ (*Pnma)* doped CaD_2_ (*Pnma*) with N_2_ at 600 ^o^C. See Supplementary Figure 7 and S8 for further details. Structural refinement was done in GSAS. The data were collected on Polaris Diffractometer at ISIS^1^.

Neutron powder diffraction data from pure calcium hydride(deuteride) was collected on Polaris Diffractometer at ISIS. The fitted pattern is shown in Supplementary Figure 7. In a separate experiment CaD_2_ was doped in situ with N_2_ (90/5/5 Ar/N_2_/H_2_, 100 cm^3^/min) at 600 °C. Datasets were collected in 2 min increments. The first 25 datasets were summed to give the pattern shown in Supplementary Figure 8. The 25^th^ dataset was the first to show the presence of the nitride-hydride(deuteride) phase. The results for the two experiments are compared in Supplementary Table 5. Two major differences between the phases are apparent. Firstly, the doped phase is significantly smaller than the pure CaD_2_ shrinking by nearly 1% (0.67%). Secondly, the hydride site fractions have decreased dramatically. Although, the doped CaD_2_ phase would contain nitride ions (N^3-^) in the hydride positions, a model using only D^-^ ions was used as the covariance resulting from the mixed occupancy sites caused the refinement to be too unstable to return a true minimum value. For charge neutrality, one N would replace 3 H ions. As the scattering cross-sections N to H are near 2:1 (5.592:11.01 barn), the overall effective scattering intensity from this site will decrease, consistent with observation. The results suggest that CaD_2_ is capable of absorbing significant amounts of nitrogen before undergoing a phase transition to the *Fd-3m* structure.


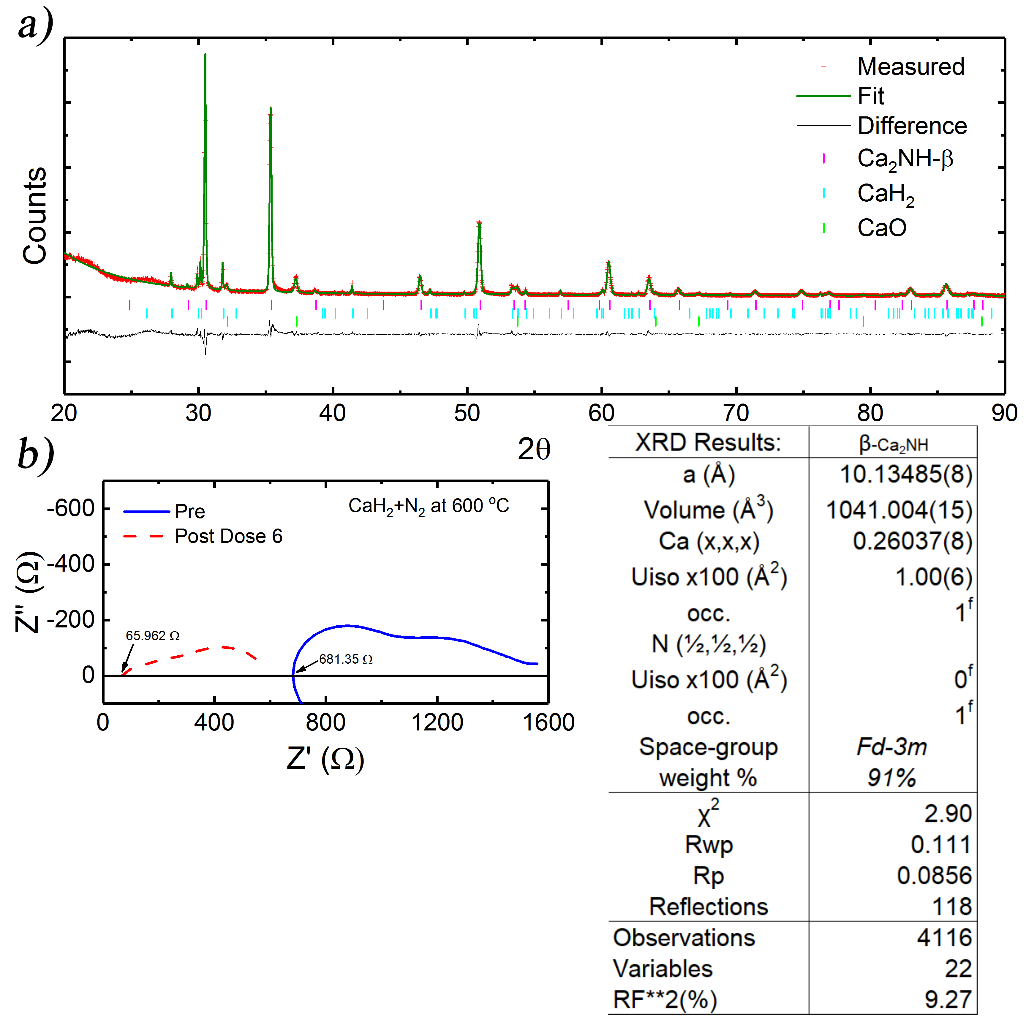


Supplementary Figure 10: Results of doping CaH_2_ with N_2_ at 600 °C. a) XRD pattern and refinement taken post experiment at RT. The pattern was collected using a PANalytical Empyrean with Cu K$\alpha$_1_ radiation between 20-90^o^ in .02^o^ increments. The data were collected over the course of an hour. Results give a 9:1 ratio for $\beta$-Ca_2_NH to CaH_2_ in agreement with the NPD results. b) EIS plot for the experiment. The *Rs* dropped from 681.35 Ω to 65.962 Ω after doping with N_2_. The model for $\beta$-Ca_2_NH was based off the published results of Brice et al.^4^. The refined structure is in close agreement with their structure. The model for CaH_2_ was from Alonso et al^10^.


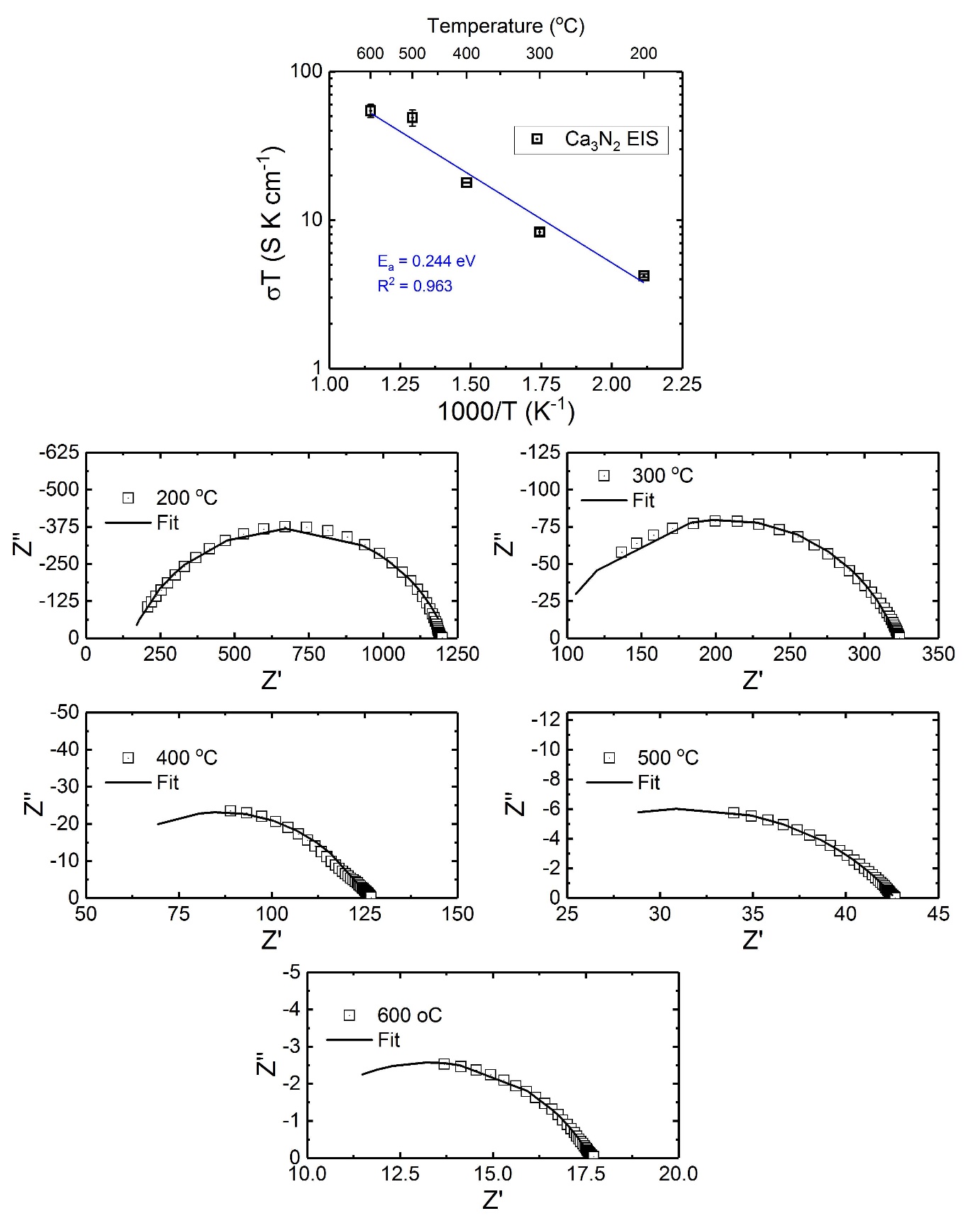


Supplementary Figure 11: An Arrhenius Plot of conductivity vs temperature for undoped α-Ca_3_N_2_ and fitted complex plane EIS data from the same experiment. The shape of the EIS curves and the activation energy from the Arrhenius plot suggest that undoped α-Ca_3_N_2_ is a semiconductor. Error bars represent one standard deviation.

Supplementary Methods:

In essence, quasi-elastic neutron scattering (QENS) measures the F.T of the root-mean squared displacement probability function. Thus, at sufficiently long times (normally limited by the counting statistics of the instrument rather than physics of the system e.g. the diffusion coefficient *D*) the quasi-elastic (near elastic but with E=/0) broadening is measureable. The momentum transfer (**Q**) dependence of this broadening provides detail on the type of dynamics being measured. The broadening of the elastic line is typically expressed in terms of half-width at half maximum ($\Gamma$) and is extracted from a convolution of a resolution function with a Lorentzian function:

$S_{inc}^{meas}\left( \mathbf{Q},\omega\right)=R\left( \mathbf{Q},\omega\right)\bigotimes\left[ A_{0}\left( \mathbf{Q} \right)\delta\left( \omega\right)+A_{1}\left( \mathbf{Q} \right)L\left( \mathbf{Q},\omega\right) \right]+B(\mathbf{Q})$ (1)

where $S_{inc}^{meas}\left( \mathbf{Q},\omega\right)$ is the measured spectra, $R\left( \mathbf{Q},\omega\right)$ is the resolution function (here taken from a measurement of the sample at 200 ^o^C where no broadening was present), $\bigotimes$ is the symbol for convolution, $A_{0}\left( Q \right)\delta\left( \omega\right)$ is static contribution function, $A_{1}\left( \mathbf{Q} \right)L\left( \mathbf{Q},\omega\right)$ is the broadening due to dynamic motion, and $B(\mathbf{Q})$ is sloped background. $\Gamma\left( \boldsymbol{Q} \right)$ is the half-width at half-maximum of $L\left( \boldsymbol{Q},\omega\right)$. $\Gamma\left( \mathbf{Q} \right)$ was fit with the Chudley-Elliott model (CEM)^11^. The CEM fits the $\Gamma\left( \mathbf{Q} \right)$ with a characteristic jump length (*l*) and time ($\tau)$. These values can be used to calculate a diffusion coefficient (*D*) according to Einstein:

$D=\frac{l^{2}}{n\tau}$ (2)

This is due to the scattering nature of 1H with neutrons which is largely incoherent (*σ_incoh_*=80.26 barn vs *σ_coh_*=1.7568 barn; 1 barn = 1x10^-24^ cm^2^). This incoherence allows for the measurement of the self-scattering function which is the double Fourier transform of the self-correlation function. The self-correlation function describes the probability of finding an atom at position *x_1_* after time *t_1_*, when said particle started at *x=0*, at *t=0*^12^. Motion of the particle over a time period manifests itself in the self scattering function as a broadening in energy (the FT of time) at specific momentum transfer (**Q**, the FT of position).

It is a well-known technique to model electrochemical impedance spectroscopy (EIS) data using equivalent circuits models (ECMs). Impedance is the alternating current equivalent of resistance. As such, impedance values have both a magnitude and phase angle. That information is encoded using complex numbers with the magnitude being given by the hypotenuse of a simple right tringle formed by the real (Z’, x-axis) and imaginary (Z”, -y-axis) components of the vector. The phase angle is given by the angle formed between the x-axis and the vector. Thus, the value of the imaginary component determines the phase angle. The final aspect of impedance to consider is that it is a function of frequency (Z(*φ*)). Thus for an EIS experiment in which many different frequencies are applied the system, the dependence of Z on *φ* tells a researcher much about the processes occurring in the system. In electrochemical systems, the charge must travel across many different regions with differing characteristics. Firstly, the different aspects of the system may have different resistances associated with charge transfer. Secondly, the different aspects have different geomeotries which give rise to characteristic capacitances associated with specific regions of the system (see Irvine et al.^13^). Thus, a specific region of the cell is characterised by its resistance and capacitance.
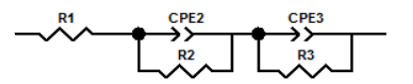
An EIS signal can thus be fit as a sum of contributing circuit components:

In this example ECM: the *series resistance* is given by R1. This value encompasses all processes that occur at rate higher than the highest frequency of the measuring machine (or at least at the same frequency of the inductance of the system. There are two RC units (resistor/capacitor in parallel), each representing a specific electrochemical process. As mentioned earlier, the value of C tells the research a lot about the specific process that has the resistance value of *R*. Here, a CPE (constant phase element) unit is used instead of a true capacitor. CPE units allow to take into account non-ideality of the capacitor unit, e.g. rather than having a single timescale associated with a process, a range of timescales can be modelled. This can arise from physical variation for the specific electrochemical process such as surface roughness.

EIS data were collected throughout the QENS experiment in 30 min increments. The sample was heated to 600 °C under flowing 5% H_2_ in Ar (100 cm^3^ min^-1^). The experiment then took on the following sequence:

1. 10 min NPD pattern was collected
2. N_2_(g) was introduced for five minutes (5/5/90 N_2_/H_2_/Ar, 100 cm^3^ min^-1^)
3. 10 min NPD pattern collected
4. 2 hr QENS dataset collected
5. Repeat

The NPD patterns were fitted using the crystal models of β-Ca_2_ND and CaD_2_+N_2_ developed from the Polaris NPD data analysis (see Supplementary Figure 9d). Due to the small number of reflections available from the Osiris diffraction bank 1, the refinements were limited to phase fractions and lattice parameters. The QENS data were fitted using the Chudley-Elliot jump-diffusion model (CEM, see Supplementary Figure 12b-c)^11^. The EIS data were fitted using equivalent circuit models as shown in Supplementary Figure 12e.


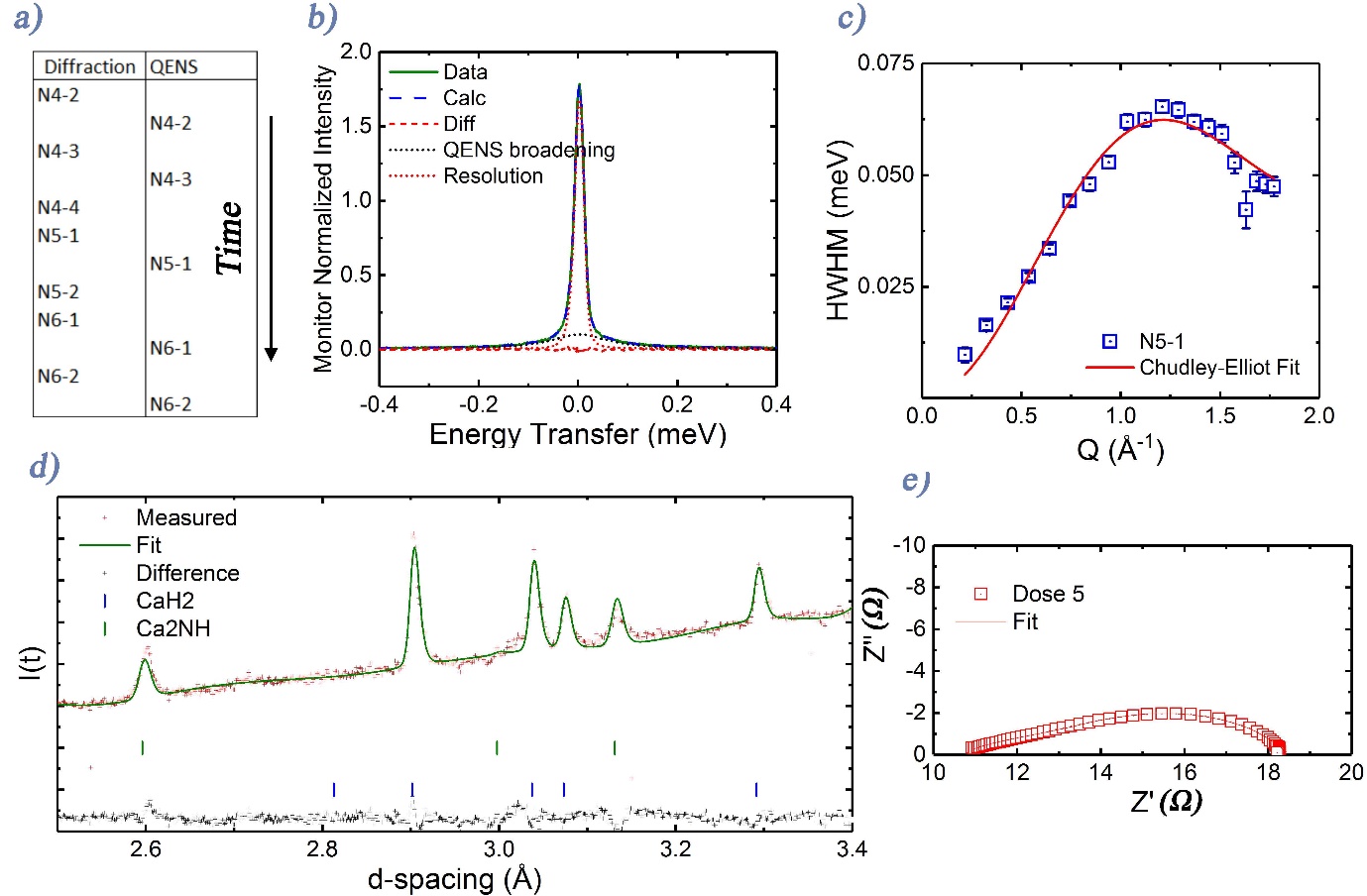


Supplementary Figure 12: Example fits from the Osiris experiment where CaH_2_ was doped with N_2_ at 600 °C. a) Table that shows the sequence of data runs. b) Fitted QENS spectrum from run N5-1, *Q* = 1.0 [Å^-1^] fit using ConvFit in Mantid. c) Chudley-Elliot Model fit result for dataset N5-1^11^. d) Diffraction pattern collected on bank 1 of the Osiris instrument fitted using cif files produced from the Polaris experiment. Results allowed for the calculation of lattice parameters and phase fractions. e) Electrochemical impedance spectroscopy complex plane plot. Plot shows the raw spectra as well as the equivalent circuits model fit. Error bars represent one standard deviation.


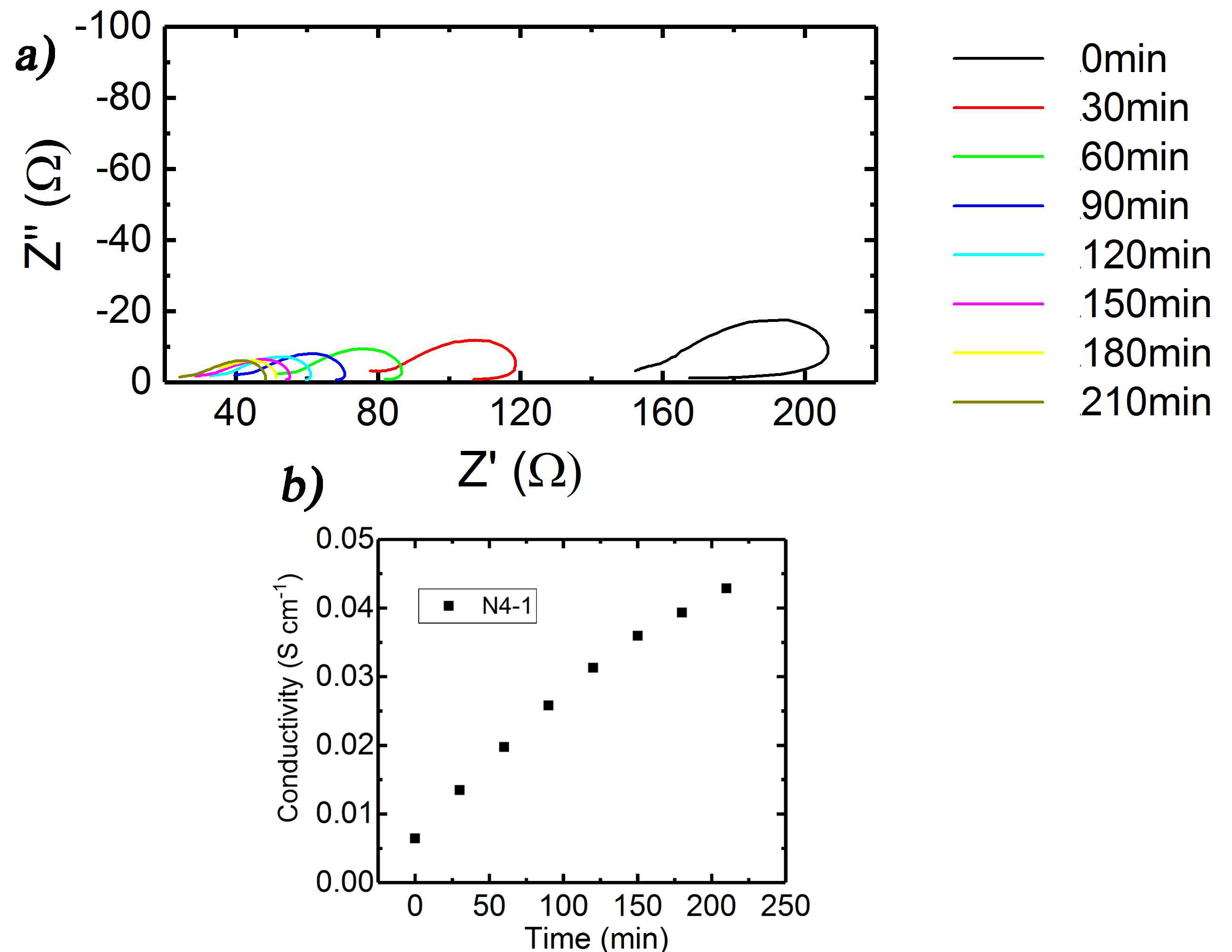


Supplementary Figure 13: Plots for dataset N4-2 collected on EIS. a) Complex plane plot of spectra collected during run show that over the course of the run, the spectra change continuously. b) Conductivity extracted from the EIS data versus time.


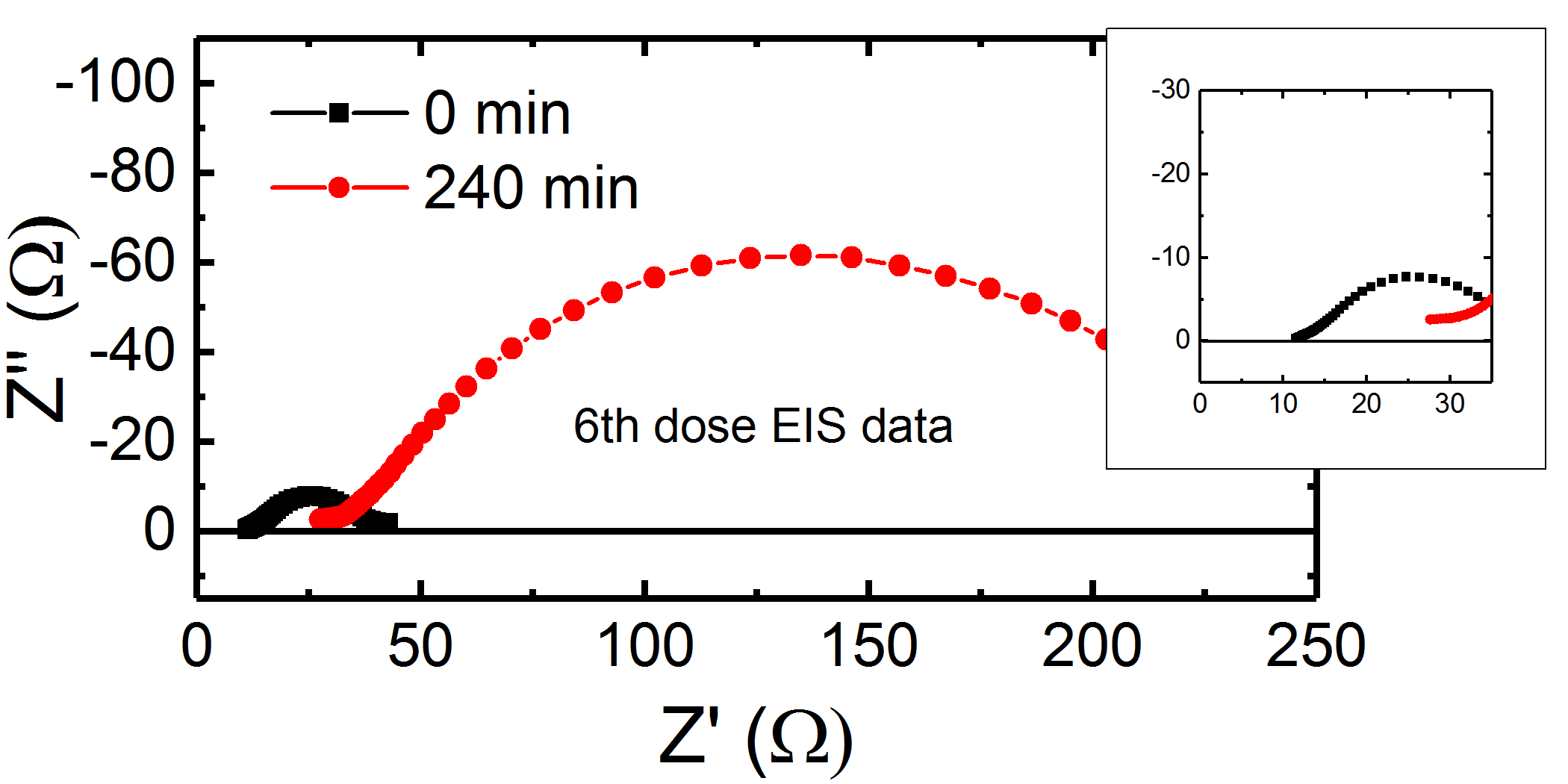


Supplementary Figure 14: Plot of EIS spectra from beginning and end of QENS run N6-1. The resistance associated with electrode-bulk interface (~10^-4^ F) grows from 23.212 Ω to 211.46 Ω. We believe this growth reflects delamination of the electrode from the pellet surface making the accurate measurement of the series resistance impossible.


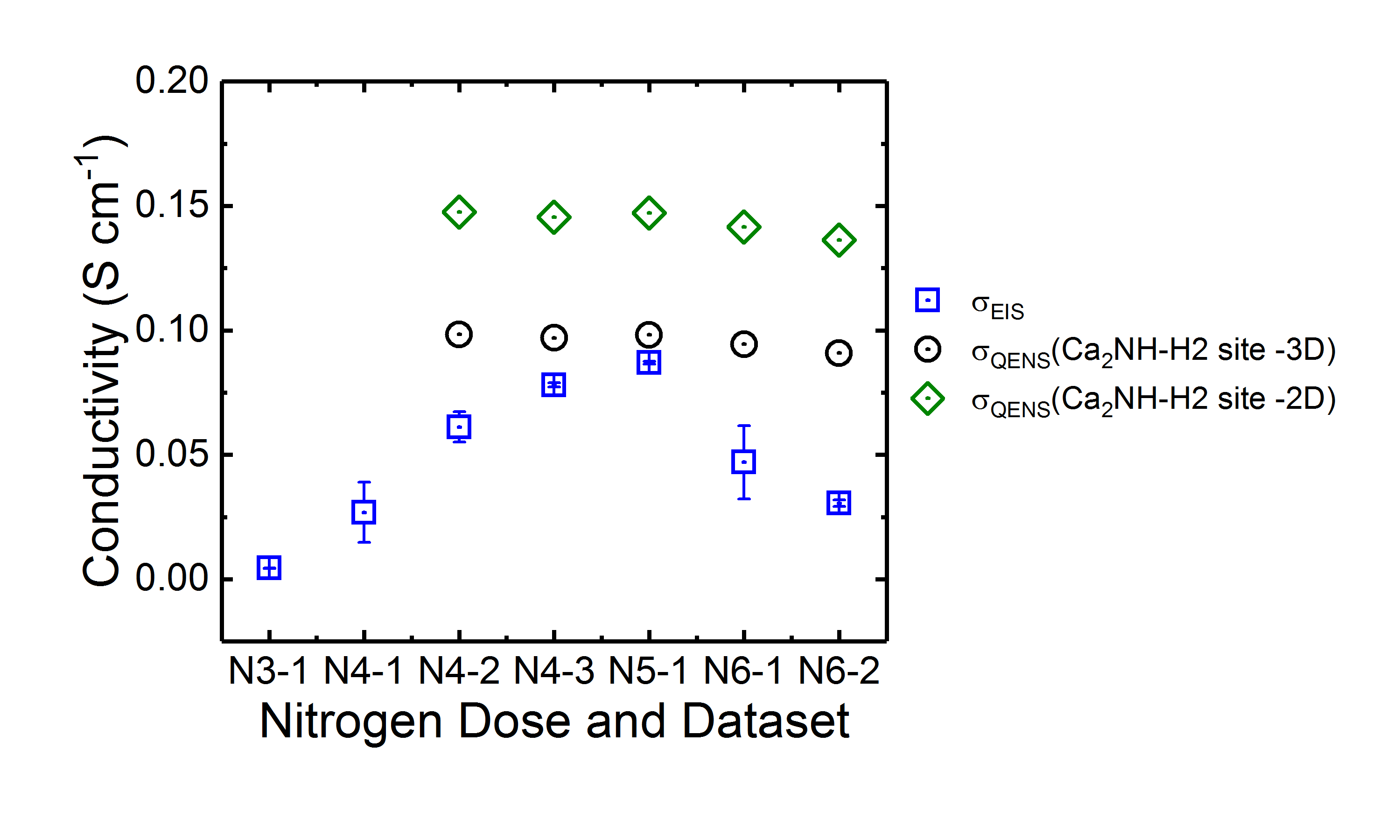


Supplementary Figure 15: Plot of conductivity calculated from the QENS data using $n_{H2}$ (tetrahedral site) for the Nernst-Einstein equation (see equation 2 of main text). Results show that using $n_{H2}$ the conductivity as measured by QENS (*σ_QENS_*) is close to that measured by EIS (*σ_EIS_*). This result led to the investigation of a vacancy mediated mechanism as, for an intrinsic vacancy creation, $n_{H2}=n_{H1,vac}$. In other words, the vacancy concentration in the main site is equal to the concentration of ions that have moved to a secondary site. Error bars represent one standard deviation.


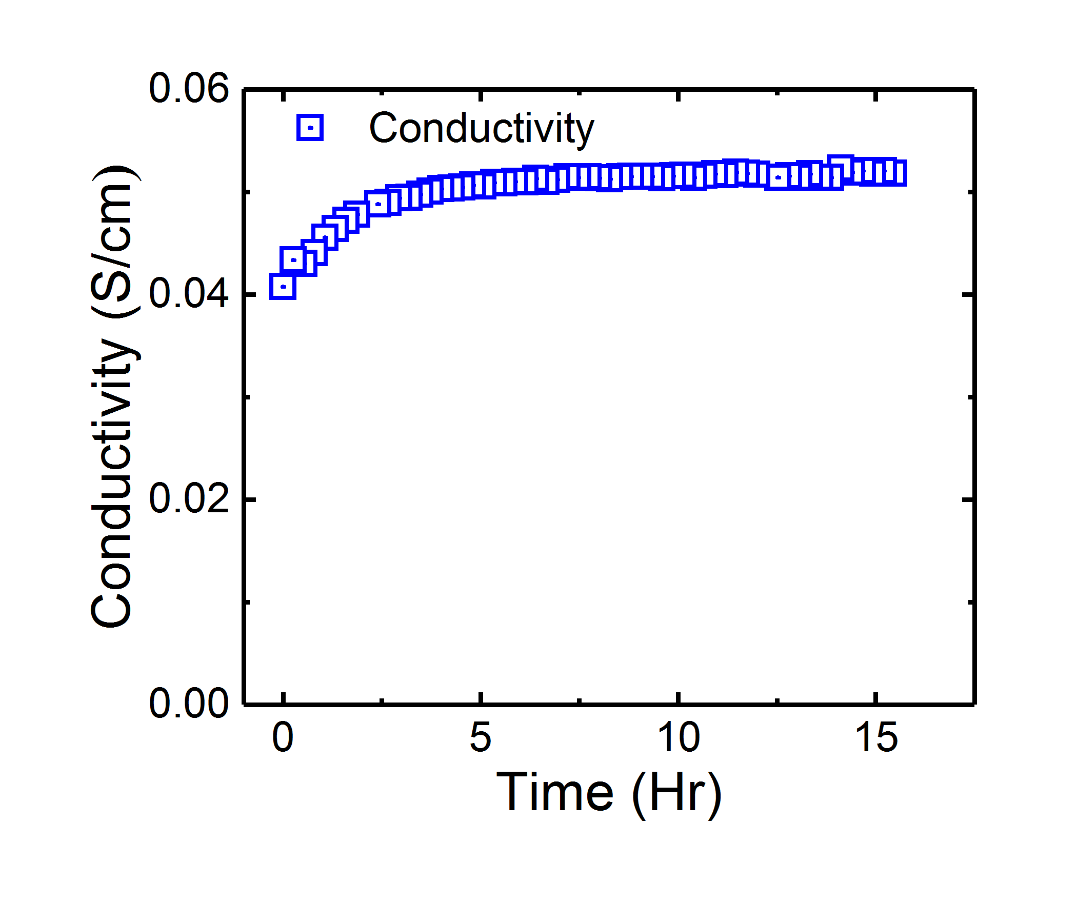


Supplementary Figure 16: Stability of β-Ca_2_NH measured over 15 hrs in flowing 5% H2 in Ar.

Data available:

CaD_2_ isotherms: <https://doi.org/10.5286/ISIS.E.53840907>

**Supplementary References:**

1. Smith, R. I. *et al.* The upgraded Polaris powder diffractometer at the ISIS neutron source. *Rev. Sci. Instrum.* **90**, (2019).

2. Reckeweg, O. & DiSalvo, F. J. About binary and ternary alkaline earth metal nitrides. *Zeitschrift für Anorg. und Allg. Chemie* **627**, 371–377 (2001).

3. Shen, C. H., Liu, R. S., Lin, J. G. & Huang, C. Y. Phase stability study of La_1.2_Ca_1.8_Mn_2_O_7_. *Mater. Res. Bull.* **36**, 1139–1148 (2001).

4. Brice, J. F., Motte, J. P., Courtois, A., Protas, J. & Aubry, J. Etude structurale de Ca2NH par diffraction des rayons X, diffraction des neutrons et resonance magnetique nuclaire du proton dans le solide. *J. Solid State Chem.* **17**, 135–142 (1976).

5. Kitano, M. *et al.* Essential role of hydride ion in ruthenium-based ammonia synthesis catalysts-SI. *Chem. Sci.* **7**, 4036–4043 (2016).

6. Verbraeken, M. C., Suard, E. & Irvine, J. T. S. Order and disorder in Ca2ND0.90H0.10 - A structural and thermal study. *J. Solid State Chem.* **184**, 2088–2096 (2011).

7. Reckeweg, O. & Di Salvo, F. J. Alkaline earth metal nitride compounds with the composition M2NX (M = Ca, Sr, Ba; X = ???, H, Cl or Br). *Solid State Sciences* vol. 4 575–584 (2002).

8. Makepeace, J. W. *et al.* Compositional flexibility in Li-N-H materials: implications for ammonia catalysis and hydrogen storage. *Phys. Chem. Chem. Phys.* **23**, 15091–15100 (2021).

9. Sichla, T. *et al.* Crystal structure determination of a strontium hydride imide nitride -Sr2(H)N/SrNH resp. Sr2(D)N/SrND - By X-ray, neutron, and synchrotron radiation. *Zeitschrift fur Anorg. und Allg. Chemie* **623**, 414–422 (1997).

10. Alonso, J. A., Retuerto, M., Sánchez-Bemtez, J. & Fernández-Díaz, M. T. Crystal structure and bond valence of CaH2 from neutron powder diffraction data. *Zeitschrift fur Krist.* (2010) doi:10.1524/zkri.2010.1258.

11. Chudley, C. T. & Elliott, R. J. Neutron scattering from a liquid on a jump diffusion model. *Proc. Phys. Soc.* **77**, 353–361 (1961).

12. Van Hove, L. Correlations in space and time and born approximation scattering in systems of interacting particles. *Phys. Rev.* (1954) doi:10.1103/PhysRev.95.249.

13. Irvine, J. T. S., Sinclair, D. C. & West, A. R. Electroceramics: characterization by impedance spectroscopy. *Adv. Mater.* **2**, 132–138 (1990).
